# Impaired organic and mineral extracellular matrix composition in early-onset osteoporosis

**DOI:** 10.1093/jbmr/zjaf159

**Published:** 2025-10-30

**Authors:** Agnes Ostertag, Bastien Léger, Eugenie Koumakis, Patrice Fardellone, Mylene Zarka, Thomas Funck-Brentano, Guillaume Mabilleau, Martine Cohen-Solal

**Affiliations:** Université Paris Cité, Bioscar INSERM U1132, Hôpital Lariboisière (APHP), F-75010 Paris, France; Université Paris Cité, Bioscar INSERM U1132, Hôpital Lariboisière (APHP), F-75010 Paris, France; Department of Rheumatology, Cochin Academic Hospital, F-75014 Paris, France; Université Paris Cité, INSERM UMR 1163, Imagine Institute, F-75015 Paris, France; Department of Rheumatology, Academic Hospital, F-80000 Amiens, France; Université Paris Cité, Bioscar INSERM U1132, Hôpital Lariboisière (APHP), F-75010 Paris, France; Université Paris Cité, Bioscar INSERM U1132, Hôpital Lariboisière (APHP), F-75010 Paris, France; Department of Rheumatology, Lariboisière Hospital, F-75010 Paris, France; Univ Angers, Nantes Université, ONIRIS, Inserm, RMeS UMR 1229, F-49000 Angers, France; CHU Angers, Departement de Pathologie Cellulaire et Tissulaire, F-49000 Angers, France; Université Paris Cité, Bioscar INSERM U1132, Hôpital Lariboisière (APHP), F-75010 Paris, France; Department of Rheumatology, Lariboisière Hospital, F-75010 Paris, France

**Keywords:** bone, osteoporosis, extracellular matrix, Raman, FTIR imaging

## Abstract

Early-onset osteoporosis (EOOP) is a rare form of primary osteoporosis defined by major skeletal fractures or low BMD that occurs in early age. However, the characteristics of the extracellular matrix that may contribute to bone fragility are unknown. We explored the microarchitecture and bone matrix composition in transiliac bone biopsies (BBs) obtained from adults with EOOP. We compared EOOP BBs to historical control BBs. Microarchitecture was measured by μCT and bone matrix composition by Raman microspectroscopy and Fourier transform infrared spectroscopy. Mechanical response of the bone matrix was investigated by nanoindentation. The contribution of each parameter was assessed by principal component analysis. We compared 18 BBs for EOOP patients (mean [SD] age 34 [8] yr, LS BMD Z-score −2.05 [1.04]) to 19 BBs for age-matched healthy individuals. Patients had vertebral fractures only (*n* = 7), peripheral fracture only (*n* = 6) and both vertebral and peripheral fractures (*n* = 3). Early-onset osteoporosis and controls had similar bone volume (bone volume/total volume, *p* = .741). As compared with controls, EOOP BBs showed lower trabecular separation (*p* = .026) and higher trabecular connectivity density (*p* < .001); cortical thickness was lower in EOOP BBs (*p* < .01). Also, GAG/Amide III and hydroxyproline/proline ratios as well as accumulation of AGEs were greater in EOOP than controls (all *p* < .0001). Moreover, tissue mineralization was lower in EOOP than controls, as shown by v_1_PO_4_/CH_2_ ratio, mineral maturity crystallinity and crystal size index (all *p* < .005). Hardness, indentation modulus and maximum load were all altered in EOOP. Principal component analysis revealed greater contribution of both the organic and mineral matrix phase at the trabecular and cortical EOOP bone rather than bone microarchitecture. The matrix composition of bone showed greater damage of the organic matrix phase and reduced mineralization in EOOP patients than controls, which may explain the high risk of fracture in EOOP patients and may differentiate EOOP from other bone diseases.

## Introduction

Early-onset osteoporosis (EOOP) is a rare form of primary osteoporosis defined by low BMD or major skeletal fractures that occur in early age. Revealed in men or premenopausal women before age 55 yr, the primary nature of osteoporosis remains unclear and is based on the exclusion of secondary causes. Bone fragility in young adults is a complex disease with multifactorial underlying mechanisms, among them a genetic susceptibility.^1^ Initially described as primary osteoporosis, several genes were found associated with low BMD or fractures in young adults the last decade, leading to the emergence of monogenic skeletal diseases characterized with low BMD or fractures. Therefore, this new identify characterized by low BMD and fractures is now called EOOP that include monogenic diseases and those without identified genes.^2^

The pathogenesis of EOOP includes a large number of mechanisms, among them the activity of bone cells, which determines the apposition of the extracellular matrix, bone mineralization, and microarchitecture, each being dependent or independent components of bone strength. Microarchitectural features measured by HR-pQCT could well predict fracture risk in a large cohort of men aged 40-96 yr, although the risk did not vary with age.^3^ In addition, trabecular and cortical microarchitecture are not different in patients with EOOP regardless of prevalent fractures, but a lower cortical density was found on histology.^4^

The mechanical properties of the extracellular matrix have emerged as a crucial determinant of fractures in common and rare diseases.^5^^,^^6^ Intrinsic properties of bone, including the organic and mineral composition, are additional factors that influence resilience to fractures. Bone microarchitecture and bone material properties may explain the occurrence of fractures in postmenopausal women with normal BMD.^7^ In addition, bone biopsies (BBs) from patients with EOOP who experienced major fractures showed the disorganization of the trabecular network and low cortical thickness, the two features being independent.^8^ Also, epigenetic and enzymatic mechanisms affect the material properties; both can be captured by Fourier transform infrared (FTIR) spectroscopy and Raman microspectroscopy. Applied to human BBs, these tools demonstrated the heterogeneity of mineral deposition in postmenopausal women with osteoporosis^9^ and the response to bone targeted-therapies.^10^^,^^11^ The analysis of the bone matrix revealed increased bone mineralization in children with osteogenesis imperfecta that may expose to a high fracture rate,^12^^,^^13^ this being is further increased by pamidronate injection.^14^ Hence, these approaches are crucial to assess bone quality and analyze the contribution of organic and mineral phases.

To better characterize the mechanism of fractures in EOOP, we explored the characteristics of the microarchitecture and matrix composition in BBs from adults with EOOP.

## Materials and methods

### Study design

We retrospectively analyzed BBs collected from 2009 to 2019 from 3 tertiary academic centers dedicated to osteoporosis, to which patients were referred because of the occurrence of major fractures at a young age. There were no other clinical or radiological signs that could have suggested any other associated disease. No patient had a history of anorexia or any disease that impaired skeletal growth. Bone biopsies were obtained from cases of non-traumatic vertebral or appendicular fractures before age 55 (EOOP; *n* = 18). Biopsies were obtained after ruling out secondary causes and before the initiation of any anti-osteoporotic treatment. Demographic, morphologic, and clinical risk factors for fracture as well as bone biomarker values were collected. Vertebral fractures were recorded with lateral radiography. At the time of the initial management, secondary causes of osteoporosis were excluded according to serum levels of creatinine to rule out renal failure, serum electrophoresis of proteins to rule out hematopoietic disease, and serum levels of calcium, phosphorus, 25OH vitamin D, alkaline phosphatase (ALP), PTH, and thyroid-stimulating hormone. To characterize the microarchitecture and bone matrix in BBs, we included a historical control group, with BBs obtained from individuals (controls; *n* = 19) who were <50 yr old, healthy, and from whom biopsies were collected for measuring internal control parameters.^15^ Controls were selected on the basis of the absence of any other disease, but no clinical bone investigation was done.^15^

### BMD and bone biomarker collection in EOOP patients

BMD was measured in EOOP patients by DXA at the LS and TH with Lunar (General Electrics) or Hologic devices. There was no cross-calibration among centers, so results are reported as T-scores and Z-scores, as recommended in clinical practice. Serum bone biomarkers were measured at the time of the bone biopsy and were measured in the morning in fasting conditions. We measured the serum levels of ALP (Atellica CH Alkaline Phosphatase ALP-2c), PTH (Atellica IM PTH, Siemens, Centaur XP, Siemens) and PTH kit assay liaison (Diasorin), and C-telopeptides (CTX; ELISA IDS and ELISA Roche). BMD parameter and blood test values were available for only EOOP patients.

### Bone biopsy

Transiliac BBs were performed under local anesthesia by using a 7.5-mm internal-diameter Bordier trephine, 2 cm below the iliac crest and 2 cm behind the antero-superior iliac crest. All specimens were fixed in ethanol, dehydrated in xylene at 4 °C, and embedded in polymethylmethacrylate without any previous decalcification.

### Microarchitecture analysis of bone biopsy cores

Microarchitectural analysis was performed by μCT analysis in the same block. The whole bone specimen was scanned by using a SkyScan 1272 scanner (Bruker μCT) with v1.5 acquisition software. The source voltage and current were 70 kV and 140 μA. All scans were obtained at 180° with image pixel size of 8 μm. To remove soft radiographs, a 0.5-mm-thick aluminum filter was placed in front of the X-ray source. The angular rotation step was fixed at 0.25°, with frame averaging 2 and exposure acquisition time per frame 1200 ms. Reference images without specimens were obtained before each acquisition for flat field correction. SkyScan volumetric NRecon reconstruction software (v1.7.4.6) was used to reconstruct cross-section slices from acquired μCT angular projections through the object. Image processing and analysis involved using Bruker CTan v1.23, giving size and microarchitecture bone parameter values*.* We measured the ratio of trabecular bone volume to total volume (BV/TV %), trabecular separation (Tb.Sp μm), trabecular thickness (Tb.Th μm), connectivity density (mm^−3^), and trabecular BMD (g.cm^−3^). Cortical analysis included the measurement of cortical thickness (Ct.Th μm) and cortical BMD (g.cm^−*3*^).

### Analysis of bone composition by FTIR spectroscopy and Raman microspectroscopy

For Raman microspectroscopy analysis, the bone specimen was surface-ground with sandpaper (Grade 2400, then Grade 4000) and surface-polished with diamond paste (Struers). Three trabecular and 2 cortical regions of interest were randomly selected in each bone specimen. For each region of interest (trabecular or cortical bone), a line was drawn across the entire width of the trabecula or cortical wall (Figure S1). Raman spectra were acquired every 1.25 μm along this line. The line intersected bone structural units of different tissue ages. For each measured parameter, a histogram distribution was then generated based on these spatially resolved measurements. Raman spectra were recorded with an inVia Qontor confocal Raman microscope with a holographic grating (1200 lines/mm) and a 785 nm laser source at 30 mW power (Renishaw) in the spectral range 800-1800 cm^−1^. Each spectrum was acquired as the sum of three consecutive spectra with each collected for 20 s with a 20X objective (NA = 0.4), focusing the laser into a ~2.4-μm spot. With this setup, the Raman system reached a spatial resolution of ~1.2 μm and a spectral resolution of ~1 cm^−1^. The Raman system was calibrated daily with an internal silicon sample to ensure wavenumber accuracy. Spectral pre-processing was performed with an in-house written script in Matlab R2021b (The MathWorks) that consisted of background fluorescence removal with a fifth-order polynomial fitting algorithm, pMMA contribution subtraction and smoothing with a Savitzky–Golay filter (degree 2, window size 17).

Before computing any Raman parameters, background signals (from resin, unmineralized bone, or soft tissue in the bone marrow space) were excluded using a thresholding routine based on the presence of the ν₁PO₄ peak. For each parameter, a histogram distribution was generated from the line ROIs by counting the number of points whose parameter values fell within each defined interval (bin). The variable *f_i_* denotes the number of counts in a given bin, and *x_i_* the corresponding parameter value. The histograms were normalized such that the sum of all bin values (*f_i_*) equaled 100%, and were plotted as line graphs connecting the values of each bin. For each biopsy, the 2 cortical (or three trabecular) ROI lines were combined into a single histogram, and the weighted mean was computed as follows:


$$ \mathrm{mean}=\frac{\sum{x}_i\times{f}_i}{100}. $$

The following physicochemical parameters were determined from spectra:

- Collagen matrix maturity, 1670/1690 intensity ratio, the intensity ratio of subpeaks located at ~1670 and ~1690 cm^−1^ and representing the collagen matrix maturity.^16^ Given that the Amide I region includes contributions from several secondary structures, the 1670/1690 ratio serves as an indicator of collagen secondary structure rather than a direct measure of crosslinking and will require further validation.
- Carboxymethyl-lysine (CML)/CH_2_ ratio, the intensity ratio between the CML peak, located at ~1150 cm^−1^, and the CH_2_-wag peak.^17^
- Pentosidine (Pen)/CH_2_ ratio, the intensity ratio between the Pen peak, located at ~1495 cm^−1^, and the CH_2_-wag peak.^17^
- GAG/Amide III ratio computed as the area ratio between the GAG peak located in the region ~1365-1390 cm^−1^ and the Amide III band located in the region ~1243-1320 cm^−1^.^18^
- Hydroxyproline/Proline (Hyp/Pro) ratio, the intensity ratio between the hydroxyproline band at ~872 cm^−1^ and the proline band at ~854 cm^−1^.^19^
- v_1_CO_3_/v_1_PO_4_, the intensity ratio between the v_1_CO_3_ peak, located at ~1070 cm^−1^ and the v1PO_4_ peak.^20^
- v_1_PO_4_/CH_2_, the intensity ratio between the ν_1_PO_4_ peak (~960 cm^−1^) and CH_2_-wag peak (~1450 cm^−1^), representing all the components of the organic bone matrix (collagen, non-collagenous proteins and lipids). This ratio correlated best with mineralization degree assessed by quantitative backscatter electron microscopy.^21^
- Mineral maturity crystallinity (MMC) computed as the inverse of the full width at half maximum intensity of the ν_1_PO_4_ band (~960 cm^−1^). This parameter is correlated with crystallite length (002 crystallographic reflection).^22^
- Nanoporosity, the area ratio between the pMMA contribution before subtraction (800-835 cm^−1^) and the v_1_PO_4_.

After Raman assessment, bone specimens were cut to 1-μm-thick section with use of a diamond knife on an ultramicrotome. Sections were deposited onto a BaF2 window, and FTIR imaging was performed as reported previously.^23^ Briefly, sections were analyzed with a Bruker Hyperion 3000 infrared microscope coupled to a Vertex 70 spectrometer by using a 64 × 64 focal plane array detector. The FTIR imaging region of interest was centered on the line analyzed by Raman microspectroscopy. The spectrometer was filled with desiccant pellets to reduce the contribution of atmospheric water and CO_2_. A 15X Cassegrain objective (NA 0.4) was used for all acquisitions. The FPA detector was cooled with liquid nitrogen for higher sensitivity. Mid-infrared spectra were recorded at resolution 4 cm^−1^ (spectral region 900-2000 cm^−1^), with 32 accumulations in transmission mode. Background spectra were also recorded with the same specifications. Post-processing of spectra involved a lab-created script written in Matlab and included Mie scattering correction, pMMA subtraction, normalization of v_1_,v_3_ PO_4_ peak to 1, and denoising using the Savitzky–Golay algorithm with degree 2 and span length 9. Quality control of each spectrum involved calculating the signal-to-noise ratio over the spectral range 1850-2000 cm^−1^ free of biological signal. Bone pixels were manually thresholded from bone marrow and resin by using the v_1_,v_3_ PO_4_ peak area. Non-bone pixels were assigned as NaN. Spectra were further subjected to a second derivative of the v1,v3 PO_4_ peak (900-1200 cm^−1^) for identifying sub-band locations. The following FTIR imaging parameters were computed for each bone spectrum:

- Acid phosphate, the area ratio of the sub-band located at ~1127 and 1096 cm^−1^.^24^
- Crystal size index, the area ratio of sub-bands located at ~1075 and ~1055 cm^−1^. This parameter reflects crystal size in 002, 211, 200, and 202 directions.^25^

Illustrations of Raman and FTIR spectra extracted from EOOP and control datasets are provided in Figures S2 and S3.

### Nanoindentation

Poly-methyl-methacrylate blocks used for Raman spectroscopy and FTIR imaging were polished to a 1-μm finish using diamond particles (Struers). Prior to nanoindentation testing, the blocks were rehydrated in saline for 24 h at 4 °C and then equilibrated to room temperature for 2 h. The area function of the indenter tip and instrument frame compliance were calibrated using a fused silica standard. Indentations were randomly placed within trabecular and cortical bone, spanning their full width, while avoiding regions near Haversian canals, osteocyte lacunae, or visible microcracks. Nanoindentation was performed using a NHT-TTX system (CSM Instruments) equipped with a Berkovich diamond tip and an optical positioning system. Indentations were made to a depth of 450 nm at a loading/unloading rate of 40 mN/min. A 15-s hold at peak load was applied to minimize creep deformation of bone tissue. Maximum load, indentation modulus, and hardness were calculated according to the method described by Oliver and Pharr.

### Statistical analysis

Data are presented as mean (SD) for quantitative variables and number (percentage) for categorical variables. To compare quantitative variables among the 2 groups (controls and EOOP), we used Student’s *t*-test in case of homoscedasticity (tested by Bartlett’s test) or Mann–Whitney test otherwise. To analyze the matrix composition, we plotted the distribution of each parameter against the bone area percentage, and the mean value of each specimen (controls and EOOP) was then calculated. The mean distribution of the group was then plotted and represented as mean (SD).

The sample size was determined based on data from our healthy control cohort. Among all measured parameters, the v_1_PO₄/CH₂ ratio showed the highest inter-individual variability, with a mean of 7.58 and a SD of 0.845. Assuming a minimal clinically important difference of 0.75, a statistical power of 80%, and a 2-sided α of .05, a minimum of 10 subjects was required to achieve adequate power. To enhance the robustness of the analysis and account for potential variability or data loss, we ultimately included 18 patients in the study.

To better understand the level of contribution of each measured parameter to discriminate individuals, we used principal component analysis. Principal component analysis is a statistical method used for dimensionality reduction while preserving as much variability as possible in the data. It transforms the data into a new coordinate system, where the greatest variances lie on the axes, called principal components. This helps to visualize high-dimensional data in 2D or 3D plots, making it easier to identify patterns and clusters. The contribution of each original feature to the principal components provided insights into which features are most influential in the dataset. We distinguished the trabecular and cortical bone compartments. Then, we included all quantitative measured parameters in relation to each of these compartments (microarchitecture, organic and mineralized components of the extracellular matrix).

All tests were 2-sided, with significance level fixed at 0.05. Data analysis involved using R v4.3.2 (The R Foundation for Statistical Computing) and Matlab R2021b.

## Results

### Clinical characteristics and microarchitectural indices

We analyzed 18 BBs from patients with a final diagnosis of EOOP. All were referred to the osteoporosis departments because of major fractures affecting vertebral or peripheral bone before age 55 yr. Complete blood test findings excluded patients with any secondary causes including hematologic or endocrine causes before the biopsy (ie, hypercortisolism, no hyperparathyroidism, no calcium or phosphate abnormalities, no osteomalacia, no celiac or thyroid disease, and no chronic kidney disease). Table 1 shows the clinical characteristics of patients. Mean (SD) age was 34 (8) yr and mean BMI was within the normal range. Fractures occurred in 15/18 patients (83%): 4 patients had femoral fracture only, 6 vertebral fractures only, 3 both vertebral and peripheral fractures, and 2 other fractures (pelvis and humerus). The mean BMD Z-score was low at the LS (−2.05 [1.04]) and was −1.40 (1.42) for the TH. Values for mineral metabolism markers were within the normal range. The control group consisted of 19 BBs (mean age 31 [8] yr), 13 men (age 32 ± 9 yr) and 6 women (age 28 ± 5 yr).

**Table 1:** Clinical, biochemical, and BMD characteristics of patients with early-onset osteoporosis (EOOP).

	Control n = 19	EOOP n = 18
**Age (yrs)**	31 (8)	34 (8)
**Women, n (%)**	6 (32%)	11 (61%)
**BMI (kg/m^2^)**	23.6 (1.4)	23.6 (4.8)
**Recent fractures (number, %)**	0	15 (83%)
**Fracture history (number, %)**	NA	5 (28%)
**Lumbar Spine Z-score**	NA	−2.05 (1.04)
**Total hip Z-score**	NA	−1.40 (1.42)
**Alkaline phosphatase (IU)**	NA	74 (27)
**25OH vitamin D (ng/ml)**	NA	36 (12)
**Parathyroid hormone (pg/ml)**	NA	42.5 (38.7)

Clinical characteristics are shown for controls and all EOOP patients, including men and women. BMI: body mass index.

**Figure 1:**
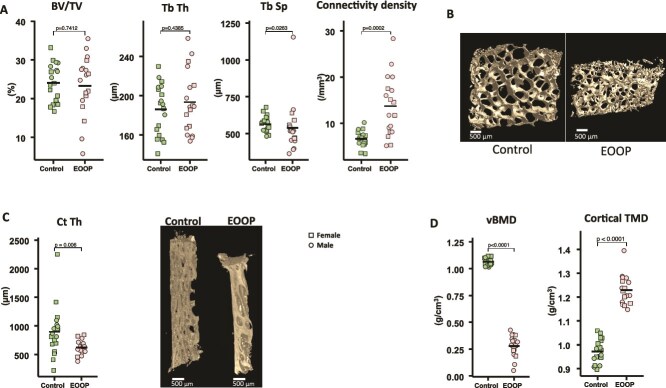
Microarchitecture analysis of bone biopsies (BBs) in early-onset osteoporosis (EOOP) and controls. Transiliac BBs measured by μCT. (A) Quantification of bone volume/total volume (BV/TV), trabecular thickness (Tb.Th), trabecular spacing (Tb.Sp), and connectivity density in controls and EOOP patients. (B) Illustration of trabecular bone in control and EOOP biopsies. (C) Quantification of cortical thickness (Ct.Th) and illustration. (D) Quantification of mineral density in trabecular (volumetric BMD [vBMD]) and cortical bone (cortical tissue mineral density [TMD]).

For patients and controls, BBs were performed before treatment with bone-influencing agents. Early-onset osteoporosis and control groups had similar bone volume (mean BV/TV: 23.3 [7.8]% vs 24.0 [4.9]%, *p* = .741) and Tb.Th (193 [31] vs 186 [26] μm, *p* = .439) (Figure 1A and B). However, EOOP biopsies had lower Tb.Sp (540.0 [172.5] vs 564.9 [54.0] μm, *p* = .026) and higher connectivity density (13.7 [6.5] vs 6.6 [1.7] mm^−3^, *p* < .001) as compared with controls (Figure 1A and B), which highlights the contribution of microarchitecture to bone strength. Mean Ct.Th was lower in EOOP than control biopsies (625.0 [125.8] vs 900.3 [429.6] μm, *p* < .01, Figure 1C). In addition, volumetric BMD (vBMD) was markedly lower in EOOP than controls at the trabecular bone (0.279 [0.099] vs 1.063 [0.035] g/cm^3^, *p* < .0001) and higher at the cortical bone (1.230 [0.971] vs 0.971 [0.049] g/cm^3^, *p* < .0001; Figure 1D).

### Impaired organic composition of extracellular matrix in EOOP

**Figure 2:**
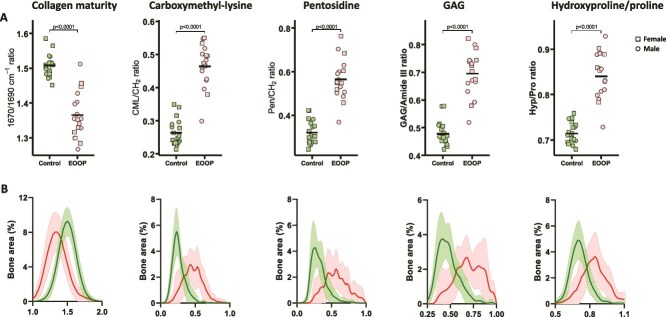
Organic composition at trabecular bone by Raman microspectroscopy. Organic matrix was analyzed at the trabecular bone. (A) Collagen matrix maturity (1670/1690 ratio), carboxymethyl-lysine level (CML/CH_2_ ratio), pentosidine level (Pen/CH_2_ ratio), GAG/Amide III ratio, and hydroxyproline/proline (Hyp/Pro) ratio plotted for controls and EOOP patients. (B) Density plots of the same parameters for controls (green lines) and EOOP patients (red lines).

**Figure 3:**
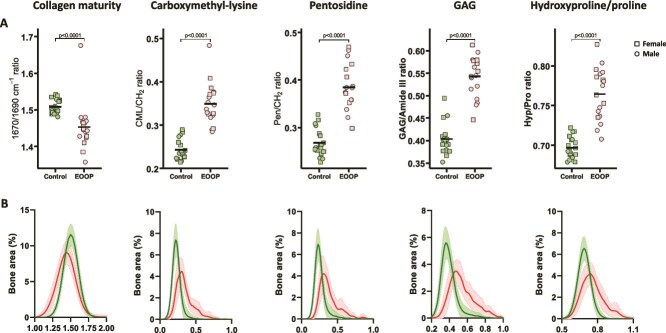
Organic composition at cortical bone by Raman microspectroscopy. Organic matrix analyzed at the cortical bone. (A) Collagen matrix maturity (1670/1690 ratio), CML/CH_2_ ratio, pen/CH_2_ ratio, GAG/Amide III ratio, and Hyp/Pro ratio are plotted for controls and EOOP patients. (B) Density plots of same parameters for controls (green lines) and EOOP patients (red lines).

We explored the organic phase of the bone extracellular matrix at trabecular and cortical bone by Raman microspectroscopy (Figures 2A, B and 3A, B). We found reduced collagen maturity, as shown by the 1670/1690 ratio (trabecular: 1.37 [0.06] vs 1.51 [0.03] cm^−1^, *p* < .0001, cortical: 1.45 [0.06] vs 1.51 [0.02] cm^−1^, *p* < .0001). In contrast, GAG/Amide III ratio was higher in EOOP than control biopsies at both the trabecular and cortical bone (trabecular: 0.70 [0.08] vs 0.48 [0.04], *p* < .0001, cortical: 0.54 [0.05] vs 0.40 [0.03], *p* < .0001) and Hyp/Pro ratio (trabecular: 0.84 [0.05] vs 0.71 [0.02], *p* < .0001, cortical: 0.76 [0.03] vs 0.70 [0.01], *p* < .0001) (Figures 2A and 3A). In addition, levels of the advanced glycation end products (AGEs) carboxymethyl-lysine (CML/CH_2_ ratio) and pentosidine (Pen/CH_2_ ratio) were significantly higher in EOOP than control biopsies at the trabecular bone (0.46 [0.06] vs 0.26 [0.04], *p* < .0001, and 0.56 [0.09] vs 0.32 [0.05], *p* < .0001, respectively) and cortical bone (0.35 [0.05] vs 0.24 [0.02], *p* < .0001, and 0.38 [0.05] vs 0.27 [0.03], *p* < .0001, respectively) (Figures 2A and 3A).

### Reduced bone mineralization in EOOP

**Figure 4:**
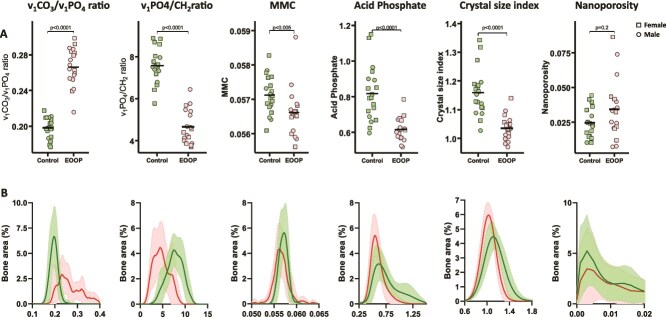
Mineral composition at trabecular bone by Raman microspectroscopy. Mineral phase analyzed at trabecular bone. (A) v_1_CO_3_/v_1_PO_4_, v_1_PO_4_/CH_2_, mineral maturity crystallinity (MMC), acid phosphate level, crystal size index, and nanoporosity for controls and EOOP patients. (B) Density plots of the same parameters for controls (green lines) and EOOP patients (red lines).

**Figure 5:**
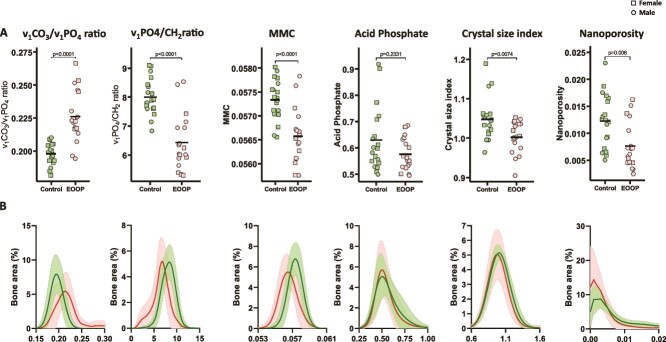
Mineral composition at cortical bone by Raman microspectroscopy. Mineral phase analyzed at cortical bone. (A) v_1_CO_3_/v_1_PO_4_, v_1_PO_4_/CH_2_, MMC, acid phosphate level, crystal size index, and nanoporosity for controls and EOOP patients. (B) Density plots of same parameters for controls (green lines) and EOOP patients (red lines).

We measured the composition of the mineral phase in trabecular and cortical bone by Raman and FTIR imaging (Figures 4A, B and 5A, B). Analysis revealed lower tissue mineralization in EOOP than control biopsies as assessed by the ratio of v_1_PO_4_/CH_2_ (trabecular: 4.7 [0.8] vs 7.6] [0.8, *p* < .0001, cortical: 6.4 [1.0] vs 8.0 [0.6], *p* < .0001) and MMC (trabecular: 0.0566 [0.0007] vs 0.0571 [0.0005], *p* < .005, cortical: 0.0566 [0.0006] vs 0.0573 [0.0004], *p* < .0001). In contrast, the v_1_CO_3_/v_1_PO_4_ ratio was enhanced at the trabecular bone (0.27 [0.02] vs 0.20 [0.01], *p* < .0001, Figure 4A) and cortical bone (0.23 [0.02] vs 0.20 [0.01], *p* < .0001, Figure 5A). Moreover, the crystal size index and acid phosphate level were significantly lower in EOOP than control biopsies at the trabecular bone (1.04 [0.04] vs 1.16 [0.08], *p* < .0001 and cortical bone 0.62 [0.07] vs 0.82 [0.15], *p* < .0001, Figures 4A and 5A).

### Reduced mechanical properties of the bone ECM in EOOP

The mechanical response of the bone extracellular matrix in trabecular and cortical bone was investigated by nanoindentation. Analysis highlighted lower values of hardness (trabecular: 142 ± 68 vs 207 ± 88, *p* = .0061; cortical: 206 ± 69 vs 310 ± 167, *p* = .0310), indentation modulus (trabecular: 5.55 ± 1.98 vs 7.34 ± 2.58, *p* = .0569; cortical: 7.79 ± 1.84 vs 10.30 ± 2.99, *p* = .0220) and maximum load (trabecular: 1.24 ± 0.27 vs 1.54 ± 0.35, *p* = .0075; cortical: 1.59 ± 0.32 vs 2.13 ± 0.75, *p* = .0050) in EOOP patients.

### Factors associated with bone fragility

**Figure 6:**
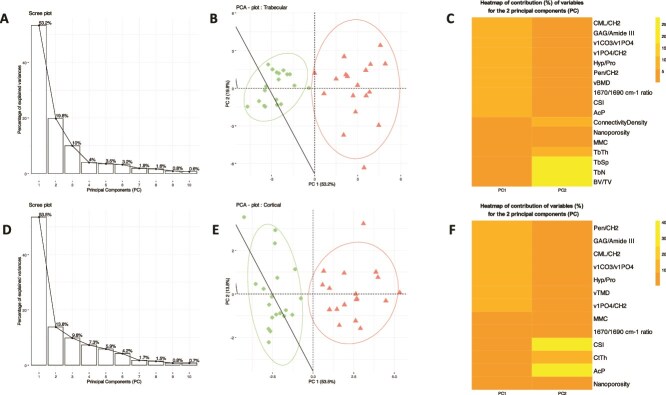
(A) Principal component analysis of bone parameters (microarchitecture, organic, and mineralized components) of the extracellular matrix for the 37 biopsies. (B) Points (controls) and triangles (EOOP).

**Figure 7:**
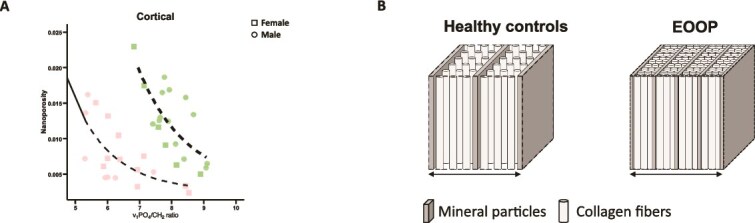
Mineral composition in EOOP bone. (A) Nanoporosity versus mineral/matrix values plotted for cortical bone. (B) Schematic representation of bone nanostructure in healthy and EOOP patients. Within a fixed volume symbolized by the horizontal arrow, the total space between collagen fibers and mineral particles is reduced, as indicated by TMD, v_1_PO_4_/CH_2_ ratio and crystal size index. The mineral particles are more numerous but thinner in EOOP bone, but the collagen fibers are also more numerous.

PCA analysis showed that two principal components (PC1 and PC2) explained 73% and 68% of the variation between individuals whatever the compartment, trabecular (Figure 6A) or cortical (Figure 6D). For each bone compartment (Figure 6B and E), 2 clusters clearly discriminated the 2 studied groups: controls and EOOP. A heatmap of the contribution of variables to PC1 and PC2 at the trabecular bone (Figure 6C) revealed that factors contributing mainly to PC1 were those concerning the mineral and organic matrix and vBMD, whereas microarchitecture parameters contributed to PC2. A heatmap of the contribution of variables to PC1 and PC2 at the cortical bone (Figure 6F) showed a contribution of organic and MMC parameters to PC1, whereas acid phosphate (AcP), crystal size index and Ct.Th contributed to PC2. In addition, despite similar mineral/matrix values in cortical bone, nanoporosity was lower in EOOP than control biopsies (Figure 7A). Thus, in EOOP, bone matrix consists of a greater number of collagen fibers and mineral particles but a smaller size of mineral particles. As illustrated in Figure 7B, within a fixed volume symbolized by the horizontal arrow, the total space between collagen fibers and mineral particles was reduced in EOOP biopsies, as indicated by tissue mineral density (TMD), v_1_PO_4_/CH_2_ ratio and crystal size index.

## Discussion

The analysis of biopsies of patients with EOOP revealed by major fractures revealed profound differences in the matrix composition: greater GAG/Amide III and Hyp/Pro ratios and AGEs levels but lower values for mineralization parameters than control biopsies. Furthermore, the mechanical response of the bone matrix was also changed with significant reductions in hardness, indentation modulus, and maximum load. However, the microarchitecture was not altered, because EOOP and control biopsies did not differ in bone volume. This observation is consistent with previous reports, finding bone volume not impaired in BBs from 9 men with EOOP who were 50 yr old, as analyzed by μCT,^26^ but higher in secondary osteoporosis as compared with EOOP.^27^ In the last report, the trabecular connectivity was high in patients with fracture when associated with risk factors. As demonstrated previously,^28^ the high connectivity density we observed in EOOP patients might contribute to the high rate of fracture as a result of failure of mechanical bone integrity. In addition, we also showed lower cortical thickness in EOOP than control biopsies, which may participate in the bone fragility despite the higher cortical bone density. Such an alteration in cortical bone may explain the high prevalence of peripheral fractures in our relatively young population.

We measured properties of extracellular matrix by Raman microspectroscopy and FTIR imaging and found several impairments in the organic phase: collagen maturity/crosslinking (1670/1690 ratio, AGE levels) or GAG content. The lower collagen maturity (1670/1690 ratio) in EOOP than control biopsies indicates a lower stabilization of the collagen fibers, as shown in postmenopausal women associated with fracture incidence.^11^ Moreover, the higher AGE levels, along with higher content of pentosidine and carboxymethyl-lysine in cortical and trabecular bone in EOOP than control biopsies, suggest damaged non-enzymatic crosslinking and lower bone turnover. Nevertheless, these 2 major AGEs of collagen fibers were previously found to alter mechanical responses by transferring the load through crosslinks rather than allowing a sliding of the tropocollagen molecules and ultimately resulting in bond breaking within the tropocollagen.^29^ At the tissue and organ levels, AGEs are associated with reduced mechanical properties.^30^^,^^31^ Moreover, AGE levels are increased in bone of conditions with compromised bone strength, such as diabetes^32^ and osteogenesis imperfecta,^33^^,^^34^ and in urine associated with vertebral fractures in postmenopausal women.^35^ Altogether, our results suggest that a compromised organic matrix exposes to reduced bone strength in EOOP.

The mineral phase of the bone matrix is another important factor contributing to bone strength. In this study, although the TMD assessed by μCT, representing the average attenuation value of bone tissue due solely to high atomic number atoms, was higher in EOOP than healthy control biopsies; the mineral/matrix ratio determined by Raman microspectroscopy was significantly lower. Tissue mineral density represents only the amount of mineral in a given voxel, whereas the mineral/matrix ratio determined by vibrational spectroscopy corresponds to the mineral content weighted by the organic matrix in the same voxel. Thus, an increase in TMD and a decrease in mineral/matrix ratio can only be explained by an increase in organic matrix content. Theoretically, this situation should be accompanied by a reduction in nanoporosity, which represents the volume occupied by interstitial fluids within the bone matrix. Indeed, in our study, nanoporosity was lower in EOOP than control biopsies. Moreover, these results raise a major point because they reveal differences from other conditions of bone fragility. The combination of Raman and FTIR imaging is crucial because results differ from osteogenesis imperfecta, in which reduced nanoporosity is also a feature. However, osteogenesis imperfecta is unambiguously characterized by a higher mineral/matrix ratio,^12^^,^^36^ which suggests that the mechanisms leading to lower nanoporosity in EOOP are different from those in osteogenesis imperfecta. This situation can be illustrated in Figure 7B, which represents a schematic of matrix voxel composition in healthy and EOOP bone, highlighting that bone material properties can differentiate osteogenesis imperfecta and EOOP.

The size and maturity/crystallinity of mineral crystals as well as carbonate content were altered in EOOP biopsies, smaller crystals having higher carbonate content and lower maturity/crystallinity. A multitude of extrinsic and intrinsic factors influence the size and composition of bone mineral crystals, but the mineral deposition process begins with the nucleation of hydroxyapatite crystals at multiple locations in the organic phase and in particular the collagen template because of the presence of non-collagenous proteins. Therefore, alterations to the organic phase of the bone matrix are thought to modify crystal growth and hence crystal size. Alterations in crystal size have been found associated with deterioration in mechanical properties.^37^ Also, despite a decrease in crystal size, the higher number of mineral particles embedded in collagen fibrils may explain the propensity to fracture.^38^

Furthermore, in the present study, we unequivocally evidenced reduction in mechanical response of the extracellular bone matrix in EOOP suggesting that changes at the extracellular bone matrix were the basis of the overall fragility observed in this condition. Principal component analysis supported these findings by showing that the first principal component (PC1) accounted for the majority of inter-individual variability and revealed two distinct clusters, which notably corresponded to the control and EOOP groups at both trabecular and cortical bone sites. Although this does not provide mechanistic insight, these data suggest that differences in organic and mineral composition, rather than microarchitecture, were the primary factors distinguishing control and EOOP subjects. Finally, these resulted in lower mechanical properties of bone matrix in EOOP as showed by nanoindentation analysis.

Our study is limited by the absence of a genetic assessment in EOOP patients. Because genetic tools were not available at the time of the BBs, we cannot rule out the genetic cause reported in genome-wide association studies^39–42^ of rare bone diseases that could have explained the unusual fracture rate in individuals at a relatively young age.^1^ However, genetic testing was performed in 4 patients because a panel was not available previously. Among the 4 tested, only one had a pathogenic *LRP5* variant. However, our study’s strength is the combination of Raman and FTIR imaging that allows for extensive characterization of the organic and mineral phases of the bone matrix in EOOP as well as the presence of a control group.

In conclusion, our study showed that EOOP is characterized by a damaged organic phase and reduced mineral phase of bone matrix composition rather than microarchitecture distribution. The impaired bone composition in EOOP requires further investigation to understand the quality of matrix and mineralization. These data highlight mechanisms of bone fragility that may differ from other bone diseases.

## Supplementary Material

Supplementary_Figures_zjaf159

## Data Availability

The data underlying this article will be shared on reasonable request to the corresponding author.
