# Supplementary material for: Impaired organic and mineral extracellular matrix composition in early-onset osteoporosis
Source: J Bone Miner Res. 2025 Oct 30;41(4):447–56. doi: 10.1093/jbmr/zjaf159 (PMC13034671; doi:10.1093/jbmr/zjaf159)

Supplementary Figure 1: Representative Raman and FTIRI regions of interest (ROIs) used to assess bone material properties in an iliac crest biopsy. Two cortical and three trabecular Raman ROIs (dashed blue lines) were randomly positioned across bone structures. Each line was divided into points spaced 1.25 μm apart, with one Raman spectrum acquired per point. The same ROIs were then centered within areas imaged by FTIRI (yellow boxes), composed of multiple fields of view acquired using a focal plane array (FPA) detector. FTIR spectra were acquired for each pixel within the box, with a spatial resolution of 2.8 μm per pixel.

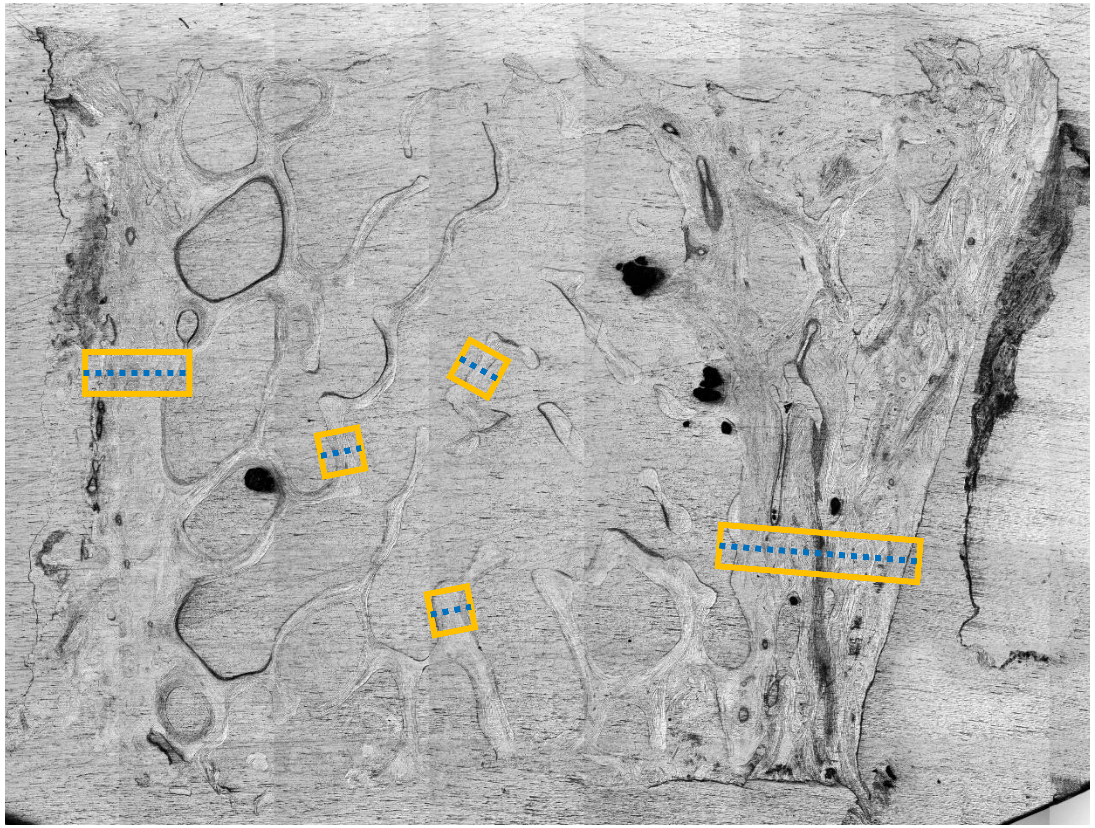

Supplementary Figure 2: Representative Raman spectra acquired from control and EOOP patients. (A) Example Raman spectra randomly selected from a line ROI in control (blue) and EOOP (red) datasets. Peaks and spectral regions used to calculate the Raman parameters are indicated. (B) For the collagen maturity parameter, the Amide I band was deconvoluted using curve fitting to extract subpeaks located at approximately 1640, 1670, and 1690 cm⁻¹. The positions of these subpeaks were identified using the second derivative of the Amide I band.

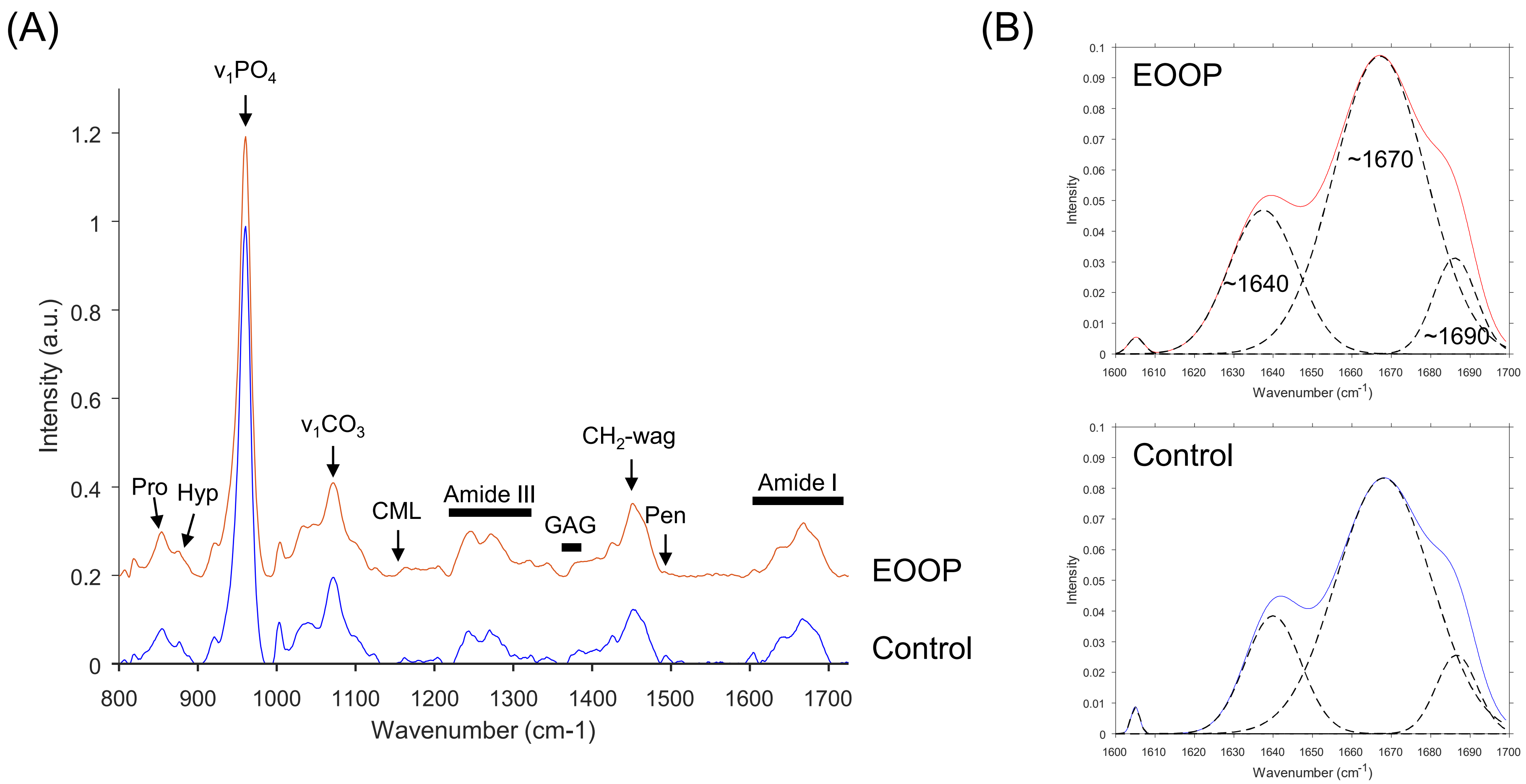

Supplementary Figure 3: Representative FTIR spectra acquired from control and EOOP patients. (A) Example FTIR spectra randomly selected from a region of interest (ROI) in control (blue) and EOOP (red) datasets. The location of the v_1,v_3PO_4 band, used to compute the acid phosphate and crystal size indices, is indicated. (B) The v_1,v_3PO_4 band was deconvoluted by curve fitting to extract subpeaks. The positions of the subpeaks were determined using the second derivative of the v_1,v_3PO_4 band. Subpeaks located at approximately 1055, 1075, 1096, and 1127 cm^{-1} were used for the calculation of acid phosphate and crystal size indices.

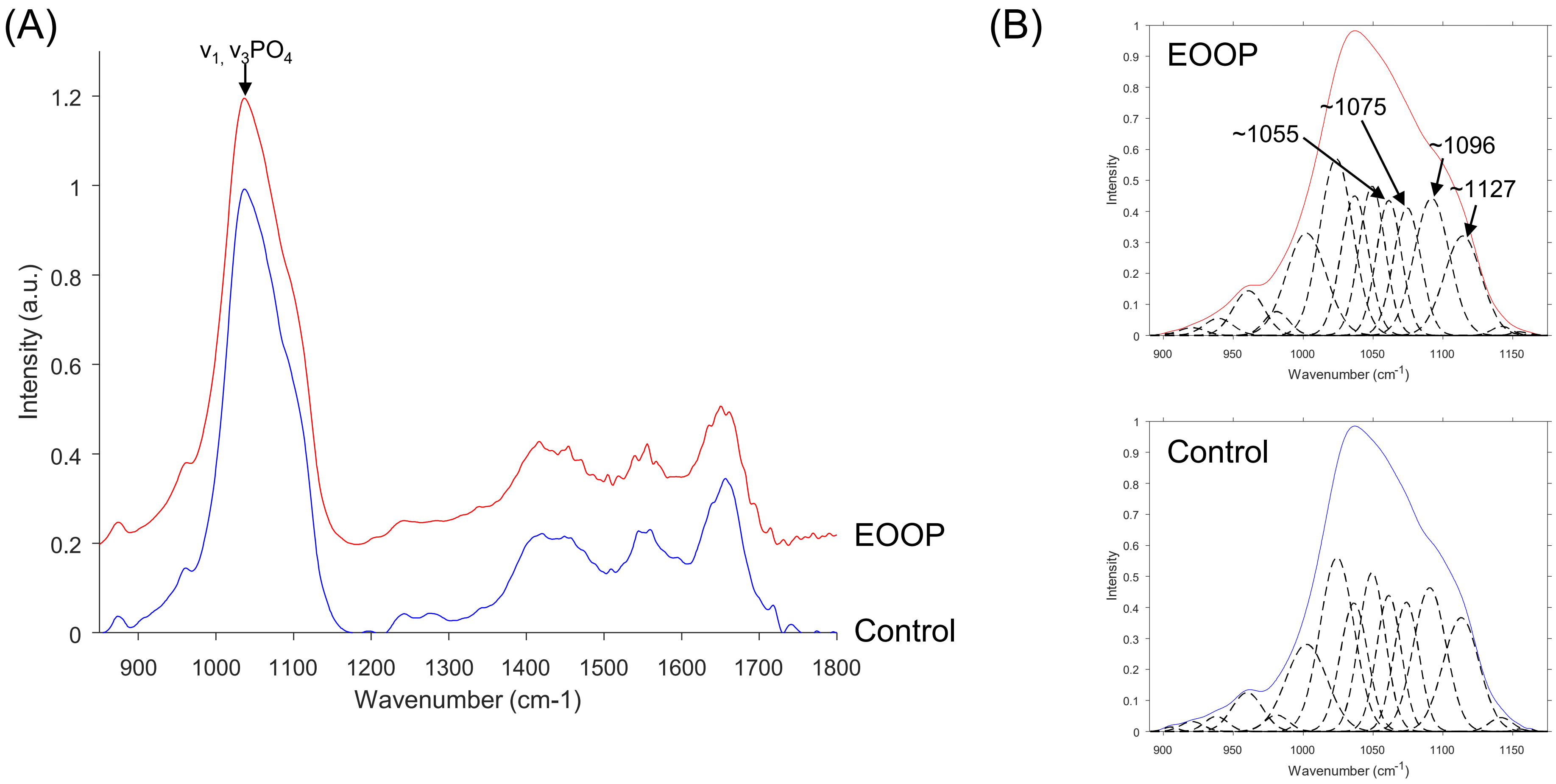

Supplement: Supplementary_Figures_zjaf159 [file supplementary_figures_zjaf159.pdf]
